# A novel anti-swing and position control method for overhead crane

**DOI:** 10.1177/0036850419883539

**Published:** 2019-11-01

**Authors:** Xuejuan Shao, Jinggang Zhang, Xueliang Zhang, Zhicheng Zhao, Zhimei Chen

**Affiliations:** 1 College of Electronic Information Engineering, Taiyuan University of Science and Technology, Taiyuan, China; 2 Shanxi Key Laboratory of Advanced Control and Intelligent Information System, Taiyuan, China; 3 College of Mechanical Engineering, Taiyuan University of Science and Technology, Taiyuan, China

**Keywords:** Takagi–Sugeno model, virtual control variable, sector nonlinear technique, linear matrix inequality, decay rate

## Abstract

Based on Takagi–Sugeno fuzzy modeling and linear matrix inequality with decay rate, this article presents a novel anti-swing and position control scheme for overhead cranes. First, the simplified nonlinear dynamic model is proposed by adopting a virtual control variable method to reduce the number of nonlinear terms. Then, the Takagi–Sugeno fuzzy model is constructed using sector nonlinear technique, and the anti-swing and position controller of overhead crane is designed based on a linear matrix inequality with decay rate. Finally, the proposed control method is compared with the traditional Takagi–Sugeno fuzzy control method, and robustness of the system is discussed. The simulation results demonstrate that the proposed method is feasible and effective.

## Introduction

Cranes belong to typical nonlinear under-actuated equipments.^1–3^ There are numerous types of cranes,^4^ such as overhead cranes, tower cranes, gantry cranes, boom cranes, and so on. With the development of productivity, cranes are widely used to transport heavy or hazardous loads in industries, harbors, construction sites, open-air storage, and so on. The overhead crane has the advantages of high flexibility of operation, strong transportation capacity, and less space occupied, so it is widely used in modern industrial production. The main purpose of overhead crane control is to deliver the load to the desired position accurately and reduce or eliminate the payload swing angle rapidly at the final position. Under the action of load inertia and external disturbance, the load oscillates inevitably in the process of transmission, which will not only reduce the efficiency of the system, but also lead to the collision between the load and the surrounding cargo or personnel. The security performance of the system is affected. Therefore, the study of crane control method has important theoretical value and practical engineering meaning.

In the past decades, research works have paid more and more attention to the problem of the anti-swinging and position control for crane systems. To improve the control performance, many control methods, which include linear control and nonlinear control, have been proposed. The linear controllers such as proportion integral derivative (PID) control,^5–8^ internal model control,^9,10^ feedback linearization control,^11–13^ input shaping control,^14–16^ and trajectory planning,^17–19^ are relatively simple and easy to be implemented. However, the linear model is established at the equilibrium point and. nonlinear uncertainties are not considered. The control method based on the model may reduce the performance of the system. With the development of nonlinear control technology, some scholars have put forward a series of nonlinear control methods based on nonlinear mathematical model. These methods include sliding model control,^2,20–22^ adaptive control,^23,24^ energy and passivity control,^25,26^ nonlinear coupling control,^27^ and model predictive control.^28–30^ However, most of these methods are too complex in design and too large in computation; in addition, a lot of nonlinear theoretical knowledge is required.

Recently, the control method based on Takagi–Sugeno (T–S) fuzzy model has been used by some scholars to study nonlinear system, such as the ball bar system,^31,32^ aircraft motion control system,^33^ inverted pendulum system,^34–36^ vehicle system,^37–39^ microhydropower plant prototype system,^40^ chaotic system,^41^ and so on. T–S fuzzy model is composed of some linear subsystems by weighted sum, which can infinitely approximate to the actual nonlinear system. Therefore, the linear system theory can be used to design controllers for each subsystem.

Constructing T–S fuzzy model has two approaches, namely local approximation,^38^ and sector nonlinearity.^34,35,37^ The former method simplifies the model by linearizing the nonlinear model at some operating points and requires fewer fuzzy rules, but the global asymptotic stability of the system cannot be guaranteed. The latter’s goal is to find the nonlinear terms and the global sector of these nonlinear terms,^42^ but it is sometimes difficult to some nonlinear systems. Based on T–S fuzzy model, many control methods are used. Wang and Fei^43^ designed a sliding mode controller for micro-electro mechanical systems. Chang et al.^44^ used an adaptive dynamic surface control for the ball and beam system. In Boulkaibet et al.,^45^ a predictive controller is given for nonlinear process.

For crane systems, there were few research works on T–S fuzzy modeling and control methods in the past. In this article, a novel T–S fuzzy modeling and control method are proposed for the overhead crane. The contributions and merits of this article include three aspects. (1) A virtual control variable method is proposed to reduce the number of nonlinear terms in crane models. T–S fuzzy model of overhead cranes is constructed based on the sector nonlinear technique. (2) In order to solve the conservative problem of linear matrix inequality (LMI), the anti-swing and position controller of overhead crane is designed based on LMI with decay rate. The controller selects the parallel distributed compensation (PDC) structure. (3)The control system has strong robustness under the condition of the trolley mass and rope length change.

The rest of this article is arranged as follows. In the second section, the T–S fuzzy model of overhead crane is constructed. The third section introduces the design of the anti-swing and position controller. The fourth section gives the numerical simulation results. Section 5 summarizes the full article.

## T–S fuzzy model of overhead crane

The simplified nonlinear model is given by adopting a virtual control variable method. The T–S fuzzy model for overhead crane is constructed based on the sector nonlinear theory in this section.

**Figure 1.**
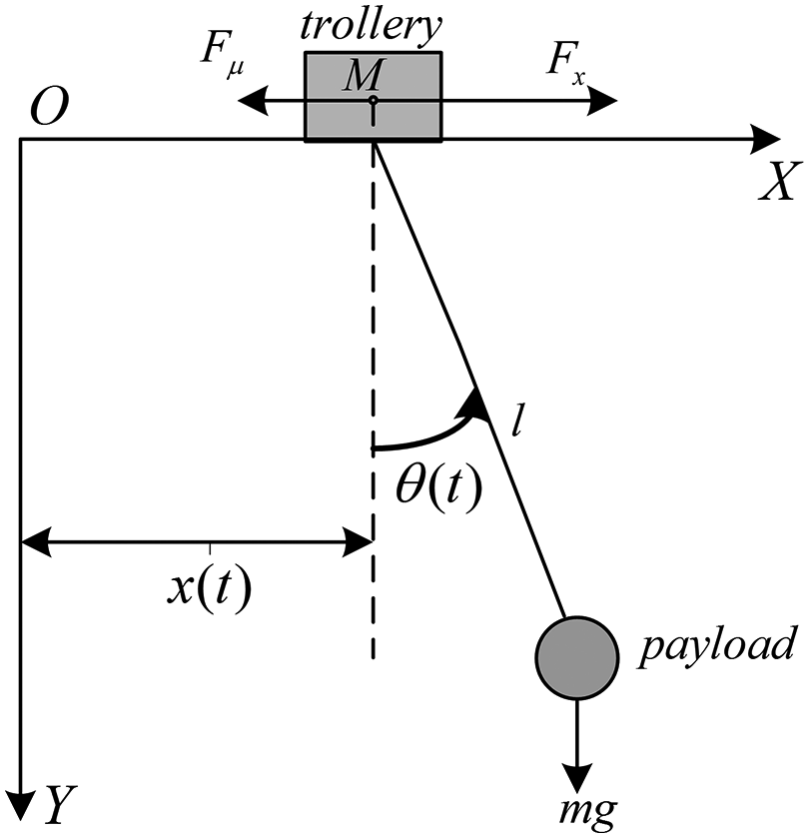
2D model of overhead crane.

The two-dimensional (2D) model of overhead crane is shown in Figure 1.

Table 1 shows the physical parametric meaning of a 2D overhead crane.

**Table 1.** The parametric meaning.

Symbols	Description	Symbols	Description
*M*	Mass of trolley	θ(t)	Swing angle of load
*m*	Mass of payload	*G*	Gravitation acceleration
x(t)	Horizontal position of trolley	$F_{x}$	Actuating force of the trolley
*l*	Length of rope	$F_{\mu }$	Horizontal friction force

Assuming that the rope length is constant, the rope mass and elasticity are not taken into account and both the trolley and the payload are treated as point mass in the derivation of dynamic model.

According to Euler–Lagrange’s equation, the dynamic nonlinear model^46^ is derived as below

(1) $$\mathrm{ml}\overset{\cdot \cdot }{\theta }\cos\theta +\left(M+m\right)\overset{\cdot \cdot }{x}-\mathrm{ml}{\overset{\cdot }{\theta }}^{2}\sin\theta +F_{\mu }=F_{x}$$

(2) $$ml^{2}\overset{\cdot \cdot }{\theta }+\mathrm{ml}\overset{\cdot \cdot }{x}\cos\theta +m\;g\;l\sin\theta =0$$

where $F_{\mu }=\mu \overset{\cdot }{x}$ , μ is the coefficient of friction, $F_{x}$ is the actuating force.

Choosing the state variables as $x=[\begin{array}{cccc} x_{1} & x_{2} & x_{3} & x_{4} \end{array}{]}^{T}=[\begin{array}{cccc} x & \overset{\cdot }{x} & \theta & \overset{\cdot }{\theta } \end{array}{]}^{T}$ , and the state equation of the overhead crane system is expressed as

(3) $$\left\{ \begin{array}{c} {\overset{\cdot }{x}}_{1}=x_{2}\\ {\overset{\cdot }{x}}_{2}=\frac{1}{\eta }\left(-\mu x_{2}+\mathrm{mg}\sin x_{3}\cos x_{3}+{\mathrm{mlx}}_{4}^{2}\sin x_{3}+F_{x}\right)\\ {\overset{\cdot }{x}}_{3}=x_{4}\\ {\overset{\cdot }{x}}_{4}=-\frac{1}{\eta l}\left(-\mu x_{2}\cos x_{3}+(M+m)g\sin x_{3}+{\mathrm{mlx}}_{4}^{2}\sin x_{3}\cos x_{3}+\cos x_{3}F_{x}\right) \end{array}\right.$$

where $\eta =(M+m)-m\;\cos^{2}x_{3}$ .

It can been see from equation (3) that the model of overhead crane is nonlinear and contains many nonlinear terms such as $\sin x_{3}\cos x_{3}$ , ${x_{4}}^{2}\sin x_{3}$ , $\cos^{2}x_{3}$ , $\sin x_{3}$ , $x_{4}^{2}\sin x_{3}\cos x_{3}$ , and so on. So, more fuzzy rules are required to construct the T–S model. To reduce the number of nonlinear terms, a virtual control variable method is used.

Suppose

(4) $$F_{x}=-\mathrm{mg}\sin x_{3}\cos x_{3}-{\mathrm{mlx}}_{4}^{2}\sin x_{3}+\left(M+m\sin^{2}x_{3}\right)u$$

in which *u* is a virtual control variable.

Equation (4) is substituted into equation (3), the nonlinear dynamic model of equation (3) is simplified to

(5) $$\left\{ \begin{array}{c} {\overset{\cdot }{x}}_{1}=x_{2}\\ {\overset{\cdot }{x}}_{2}=\frac{\mu lx_{2}}{-\eta l}+u\\ {\overset{\cdot }{x}}_{3}=x_{4}\\ {\overset{\cdot }{x}}_{4}=\frac{g\eta \sin x_{3}-\mu x_{2}\cos x_{3}+\eta u\cos x_{3}}{-\eta l} \end{array}\right.$$

According to equation (5), the premise variables $z_{1}$ , $z_{2}$ , and $z_{3}$ are defined as, respectively

$$z_{1}=\sin x_{3},z_{2}=\cos x_{3},\begin{array}{c} z_{3}=-\frac{1}{\eta l} \end{array}$$

Suppose $-\theta_{\max}\leq \theta (t)\leq \theta_{\max}$ , $-\theta_{v\max}\leq \overset{\cdot }{\theta }(t)\leq \theta_{v\max}$ , and then the membership functions of the premise variables can be obtained from the following equations

(6) $$z_{1}(t)=\sum_{i=1}^{2}\left(M_{i}(z_{1}(t))d_{i})x_{3}(t)\right)$$

(7) $$M_{1}(z_{1}(t))+M_{2}(z_{1}(t))=1$$

(8) $$z_{2}(t)=\sum_{n=1}^{2}S_{n}(z_{2}(t))b_{n}$$

(9) $$S_{1}(z_{2}(t))+S_{2}(z_{2}(t))=1$$

(10) $$z_{3}(t)=\sum_{k=1}^{2}R_{k}(z_{3}(t))f_{k}$$

(11) $$R_{1}(z_{3}(t))+R_{2}(z_{3}(t))=1$$

where $M_{i}$ , $S_{n}$ , and $R_{k}$ are member functions of the premise variables $z_{1}(t),\;z_{2}(t)$ , and $z_{3}(t)$ , respectively, and $d_{1}=d_{\max}=1,$ $d_{2}=d_{\min}=(1/\theta_{\max})\sin\theta_{\max},$ $b_{1}=b_{\max}=1,$ and $b_{2}=b_{\min}=\cos\theta_{\max}$

$$f_{1}=f_{\max}=\frac{1}{\mathrm{ml}\cos^{2}\theta_{\max}-(M+m)l},\;f_{2}=f_{\min}=\frac{1}{-\mathrm{Ml}}$$

According to equations (6)–(11), the membership functions can be calculated as

(12) $$M_{1}(z_{1}(t))=\left\{ \begin{array}{cc} \frac{z_{1}(t)-\frac{1}{\theta_{\max}}\sin\theta_{\max}\sin^{-1}(z_{1}(t))}{1-\frac{1}{\theta_{\max}}\sin\theta_{\max}\sin^{-1}(z_{1}(t))} & \mathrm{if}\;z_{1}(t)\neq 0\\ 1 & \mathrm{otherwise} \end{array}\right.$$

(13) $$M_{2}(z_{1}(t))=1-M_{1}(z_{1}(t))$$

(14) $$S_{1}(z_{2}(t))=\frac{z_{1}(t)-b_{2}}{b_{1}-b_{2}}$$

(15) $$S_{2}(z_{2}(t))=1-S_{1}(z_{2}(t))$$

(16) $$R_{1}(z_{3}(t))=\frac{z_{3}(t)-f_{2}}{f_{1}-f_{2}}$$

(17) $$R_{2}(z_{3}(t))=1-R_{1}(z_{3}(t))$$

**Figure 2.**
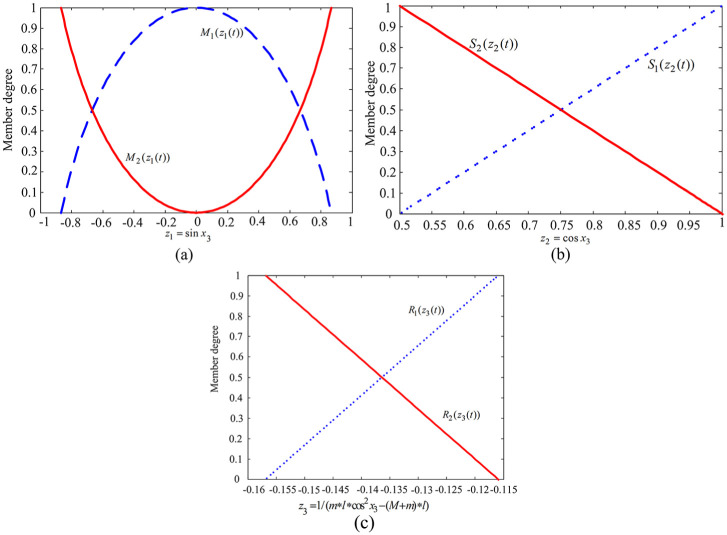
Membership function of the premise variables. (a) Member functions *M*_1_(*z*_1_(*t*)) and *M*_2_(*z*_1_(*t*)), (b) Member functions *S*_1_(*z*_2_(*t*)) and *S*_2_(*z*_2_(*t*)), and (c) Member functions *R*_1_(*z*_3_(*t*)) and *S*_2_(*z*_3_(*t*)).

The membership function diagram for $z_{1}(t),z_{2}(t)$ , and $z_{3}(t)$ is given in Figure 2.

### T–S fuzzy model

The T–S fuzzy model of nonlinear system can be approximated by a number of linear models

(18) $$\overset{\cdot }{x}(t)=\sum_{j=1}^{r}p_{j}(z(t))[A_{j}x(t)+B_{j}u(t)]$$

where

(19) $$\sum_{j=1}^{r}p_{j}\left(z(t)\right)=\frac{\sum_{j=1}^{r}\omega_{j}\left(z(t)\right)}{\sum_{j=1}^{r}\omega_{j}\left(z(t)\right)}$$

$\omega_{j}$ is the weight of rule *j*, z(t) is the premise variable, $A_{j}$ and $B_{j}$ are the system matrix and the input matrix, respectively, $p_{j}(t)$ satisfies

(20) $$\sum_{j=1}^{r}p_{j}(z(t))=1$$

As can be seen from equations (5) to (11), $z_{1}(t)$ , $z_{2}(t)$ , and $z_{3}(t)$ can be rewritten as

$$z_{1}(t)=\sin x_{3}=\sum_{i=1}^{2}(M_{i}(z_{1}(t))d_{i})x_{3}(t)$$

$$z_{2}(t)=\cos x_{3}=\sum_{n=1}^{2}S_{n}(z_{2}(t))b_{n}$$

$$z_{3}(t)=-\frac{1}{\eta l}=\sum_{k=1}^{2}R_{k}(z_{3}(t))f_{k}$$

and

$${\overset{\cdot }{x}}_{2}=z_{3}\cdot \mu lx_{2}+u$$

$${\overset{\cdot }{x}}_{4}=-\frac{g\cdot z_{1}}{l}+\mu \cdot z_{2}\cdot z_{3}\cdot x_{2}-\frac{z_{2}}{l}u$$

So, according to equations (12)–(17), T–S fuzzy model for overhead crane is written as

(21) $$\begin{array}{c} \left[\begin{array}{c} {\overset{\cdot }{x}}_{1}\\ {\overset{\cdot }{x}}_{2}\\ {\overset{\cdot }{x}}_{3}\\ {\overset{\cdot }{x}}_{4} \end{array}\right]=\sum_{i=1}^{2}\sum_{n=1}^{2}\sum_{k=1}^{2}M_{i}(z_{1}(t))S_{n}(z_{2}(t))R_{k}(z_{3}(t))\\ \;\times \left[\begin{array}{cccc} 0 & 1 & 0 & 0\\ 0 & f_{k}\cdot \mu l & 0 & 0\\ 0 & 0 & 0 & 1\\ 0 & \mu \cdot f_{k}\cdot b_{n} & -\frac{g}{l}\cdot d_{i} & 0 \end{array}\right]\left[\begin{array}{c} x_{1}\\ x_{2}\\ x_{3}\\ x_{4} \end{array}\right]+\left[\begin{array}{c} 0\\ 1\\ 0\\ \frac{1}{-l}\cdot b_{n} \end{array}\right]u\\ =\sum_{i=1}^{2}\sum_{n=1}^{2}\sum_{k=1}^{2}M_{i}(z_{1}(t))S_{n}(z_{2}(t))R_{k}(z_{3}(t))\times \left\{A_{\mathrm{ink}}x(t)+B_{\mathrm{ink}}u(t)\right\}\\ =\sum_{\beta =1}^{8}p_{\beta }(z(t))\left\{A_{\beta }x(t)+B_{\beta }u(t)\right\} \end{array}$$

where $M_{i}$ , $S_{n}$ , and $R_{k}$ are member functions of the premise variables $z_{1}(t),z_{2}(t)$ , and $z_{3}(t)$ , respectively, $A_{\beta }=A_{\mathrm{ink}}$ and $B_{\beta }=B_{\mathrm{ink}}$ .

Equation (21) means that $2^{3}=8$ rules are consisted in the T–S fuzzy model, which is shown in following Table 2. The form of each rule is as follows:

**Table 2.** Rule base for fuzzy model.

Rules	Premise variables			Linear model	Rules	Premise variables			Linear model
	$z_{1}$	$z_{2}$	$z_{3}$			$z_{1}$	$z_{2}$	$z_{3}$	
1	$M_{1}$	$S_{1}$	$R_{1}$	$\overset{\cdot }{x}=A_{1}x(t)+B_{1}u(t)$	5	$M_{2}$	$S_{1}$	$R_{1}$	$\overset{\cdot }{x}=A_{5}x(t)+B_{5}u(t)$
2	$M_{1}$	$S_{1}$	$R_{2}$	$\overset{\cdot }{x}=A_{2}x(t)+B_{2}u(t)$	6	$M_{2}$	$S_{1}$	$R_{2}$	$\overset{\cdot }{x}=A_{6}x(t)+B_{6}u(t)$
3	$M_{1}$	$S_{2}$	$R_{1}$	$\overset{\cdot }{x}=A_{3}x(t)+B_{3}u(t)$	7	$M_{2}$	$S_{2}$	$R_{1}$	$\overset{\cdot }{x}=A_{7}x(t)+B_{7}u(t)$
4	$M_{1}$	$S_{2}$	$R_{2}$	$\overset{\cdot }{x}=A_{4}x(t)+B_{4}u(t)$	8	$M_{2}$	$S_{2}$	$R_{2}$	$\overset{\cdot }{x}=A_{8}x(t)+B_{8}u(t)$

If $Z_{1}(t)$ is $M_{i}$ , $Z_{2}(t)$ is $S_{n}$ , and $Z_{3}(t)$ is $R_{k}$ , then $\overset{\cdot }{x}=A_{\beta }x(t)+B_{\beta }u(t)$ .

In Table 2, each linear model is called a “subsystem.” The system matrices can be represented, respectively, as

$$\begin{array}{c} \begin{array}{cc} A_{1}=\left[\begin{array}{cccc} 0 & 1 & 0 & 0\\ 0 & f_{1}\cdot \mu l & 0 & 0\\ 0 & 0 & 0 & 1\\ 0 & -\mu \cdot f_{1}\cdot b_{1} & -\frac{g}{l}\cdot d_{1} & 0 \end{array}\right], & B_{1}=\left[\begin{array}{c} 0\\ 1\\ 0\\ -\frac{1}{l}.b_{1} \end{array}\right],\\ A_{2}=\left[\begin{array}{cccc} 0 & 1 & 0 & 0\\ 0 & f_{2}.\mu l & 0 & 0\\ 0 & 0 & 0 & 1\\ 0 & -\mu .f_{2}.b_{1} & -\frac{g}{l}.d_{1} & 0 \end{array}\right], & B_{2}=\left[\begin{array}{c} 0\\ 1\\ 0\\ -\frac{1}{l}.b_{1} \end{array}\right] \end{array}\\ \begin{array}{cc} A_{3}=\left[\begin{array}{cccc} 0 & 1 & 0 & 0\\ 0 & f_{1}\cdot \mu l & 0 & 0\\ 0 & 0 & 0 & 1\\ 0 & -\mu \cdot f_{1}\cdot b_{2} & -\frac{g}{l}\cdot d_{1} & 0 \end{array}\right], & B_{3}=\left[\begin{array}{c} 0\\ 1\\ 0\\ -\frac{1}{l}\cdot b_{2} \end{array}\right],\\ A_{4}=\left[\begin{array}{cccc} 0 & 1 & 0 & 0\\ 0 & f_{2}\cdot \mu l & 0 & 0\\ 0 & 0 & 0 & 1\\ 0 & -\mu \cdot f_{2}\cdot b_{2} & -\frac{g}{l}d_{1} & 0 \end{array}\right], & B_{4}=\left[\begin{array}{c} 0\\ 1\\ 0\\ -\frac{1}{l}\cdot b_{2} \end{array}\right] \end{array}\\ \begin{array}{cc} A_{5}=\left[\begin{array}{cccc} 0 & 1 & 0 & 0\\ 0 & f_{1}\cdot \mu l & 0 & 0\\ 0 & 0 & 0 & 1\\ 0 & -\mu \cdot f_{1}\cdot b_{1} & -\frac{g}{l}\cdot d_{2} & 0 \end{array}\right], & B_{5}=\left[\begin{array}{c} 0\\ 1\\ 0\\ -\frac{1}{l}\cdot b_{1} \end{array}\right],\\ A_{6}=\left[\begin{array}{cccc} 0 & 1 & 0 & 0\\ 0 & f_{2}\cdot \mu l & 0 & 0\\ 0 & 0 & 0 & 1\\ 0 & -\mu \cdot f_{2}\cdot b_{1} & -\frac{g}{l}\cdot d_{2} & 0 \end{array}\right], & B_{6}=\left[\begin{array}{c} 0\\ 1\\ 0\\ -\frac{1}{l}\cdot b_{1} \end{array}\right] \end{array}\\ \begin{array}{cc} A_{7}=\left[\begin{array}{cccc} 0 & 1 & 0 & 0\\ 0 & f_{1}\cdot \mu l & 0 & 0\\ 0 & 0 & 0 & 1\\ 0 & -\mu \cdot f_{1}\cdot b_{2} & -\frac{g}{l}\cdot d_{2} & 0 \end{array}\right], & B_{7}=\left[\begin{array}{c} 0\\ 1\\ 0\\ -\frac{1}{l}\cdot b_{2} \end{array}\right],\\ A_{8}=\left[\begin{array}{cccc} 0 & 1 & 0 & 0\\ 0 & f_{2}\cdot \mu l & 0 & 0\\ 0 & 0 & 0 & 1\\ 0 & -\mu \cdot f_{2}\cdot b_{2} & -\frac{g}{l}\cdot d_{2} & 0 \end{array}\right], & B_{8}=\left[\begin{array}{c} 0\\ 1\\ 0\\ -\frac{1}{l}\cdot b_{2} \end{array}\right] \end{array} \end{array}$$

## Controller design

In this section, a LMI method with decay rate is given. The controller adopts PDC structure and the feedback gain matrices are calculated using LMI with decay rate.

Let $x_{d}$ be the desired position of the trolley and $x_{1}-x_{d}$ be position deviation. Suppose

(22) $$x_{m}={\left[\begin{array}{cccc} x_{1}-x_{d} & x_{2} & x_{3} & x_{4} \end{array}\right]}^{T}$$

To enable the trolley to reach the desired position, the control rules are as follows:

If $z_{1}(t)$ is $M_{i}$ , $z_{2}(t)$ is $S_{n}$ , and $z_{3}(t)$ is $R_{k}$

then $u_{j}(t)=-K_{j}x_{m}\;j=1,2,\ldots ,r,\;r=8$

The control law is

(23) $$u(t)=-\sum_{j=1}^{8}p_{j}(z(t))K_{j}x_{m}$$

where $K_{j}$ is the feedback gain.

*u* is a virtual control variable and cannot be directly applied to the crane system as a control signal. It is necessary to substitute equation (23) into equation (4) to calculate the control force *F*.

Substituting equation (23) into equation (18), the equation of closed loop system is yielded as

(24) $$\begin{array}{cc} \overset{\cdot }{x} & =\sum_{i=1}^{8}\sum_{j=1}^{8}p_{i}(z(t))p_{j}(z(t))G_{\mathrm{ij}}x_{m}(t)\\ & =\sum_{i=1}^{8}p_{i}(z(t))p_{i}(z(t))G_{\mathrm{ii}}x_{m}(t)+2\sum_{i=1}^{8}\sum_{i<j}p_{i}(z(t))p_{j}(z(t))\left\{\frac{G_{\mathrm{ij}}+G_{\mathrm{ji}}}{2}\right\}x_{m}(t) \end{array}$$

where $G_{\mathrm{ij}}=A_{i}-K_{j}B_{i}$

When the feedback gain is calculated by LMI, the gain value is fixed. Because of the conservatism of LMI, the control effect is poor. To improve the control effect, a LMI method with decay rate α is used. The response speed of the system is closely related to the decay rate α . Two theorems for calculating the feedback gain are given below.

### Theorem 1

The condition that fuzzy control system (24) is globally asymptotically stable is that if there exists a positive definite matrix *P*, which makes the following inequalities valid

(25) $$G_{\mathrm{ii}}^{T}P+PG_{\mathrm{ii}}<0$$

(26) $${\left(\frac{G_{\mathrm{ij}}+G_{\mathrm{ji}}}{2}\right)}^{T}P+P\left(\frac{G_{\mathrm{ij}}+G_{\mathrm{ji}}}{2}\right)\leq 0\;i<j,s\cdot t\;h_{i}\cap h_{j}\neq \phi$$

### Proof

Consider Lyapunov function $V(x(t))=x^{T}(t)\mathrm{Px}(t)$ , and then

$$\begin{array}{cc} \overset{\cdot }{V}(x(t)) & =\sum_{i=1}^{r}\sum_{j=1}^{r}p_{i}(z(t))p_{j}(z(t))x^{T}(t)\times [(A_{i}-B_{i}F_{j}{)}^{T}P+P(A_{i}-B_{i}F_{j})]x(t)\\ & =\sum_{i=1}^{r}{p_{i}}^{2}(z(t))x^{T}(t)[G_{\mathrm{ii}}^{T}P+PG_{\mathrm{ii}}]x(t)+\sum_{i=1}^{r}\sum_{i<j}2p_{i}(z(t))p_{j}(z(t))x^{T}(t)\\ & \;\times \left[{\left(\frac{G_{\mathrm{ij}}+G_{\mathrm{ji}}}{2}\right)}^{T}P+P\left(\frac{G_{\mathrm{ij}}+G_{\mathrm{ji}}}{2}\right)\right]x(t) \end{array}$$

If conditions (25) and (26) hold, $\overset{\cdot }{V}(x(t))<0$ at x(t)≠0 .

The above inequalities do not belong to LMI because of the product of unknown matrix or vector variables. The left and right of inequalities (25) and (26) are multiplied by $P^{-1}$ , respectively. Let $X=P^{-1}$ and $Q_{i}=K_{i}X$ , the following LMI conditions can be obtained

(27) $$X{A_{i}}^{T}+A_{i}X-{Q_{i}}^{T}{B_{i}}^{T}-B_{i}Q_{i}<0,$$

(28) $$X{A_{i}}^{T}+A_{i}X+X{A_{j}}^{T}+A_{j}X-{Q_{i}}^{T}{B_{j}}^{T}-B_{i}Q_{j}-{Q_{j}}^{T}{B_{i}}^{T}-B_{j}Q_{i}\leq 0.$$

### Theorem 2

The condition that $\overset{\cdot }{V}(x(t))<-2\alpha V(x(t))$ for all trajectories is equivalent to the following inequalities

(29) $$G_{\mathrm{ii}}^{T}P+PG_{\mathrm{ii}}+2\alpha P<0\;\begin{array}{c} \alpha \end{array}>0$$

(30) $${\left(\frac{G_{\mathrm{ij}}+G_{\mathrm{ji}}}{2}\right)}^{T}P+P\left(\frac{G_{\mathrm{ij}}+G_{\mathrm{ji}}}{2}\right)+2\alpha P\leq 0\;\begin{array}{c} i<j \end{array},s\cdot t\;h_{i}\cap h_{j}\neq \phi$$

### Proof

Considering Lyapunov function $V(x(t))=x^{T}(t)\mathrm{Px}(t)$ and equation (20), the condition can be written as

$$\begin{array}{c} \overset{\cdot }{V}(x(t))+2\alpha V(x(t))=\sum_{i=1}^{r}\sum_{j=1}^{r}p_{i}(z(t))p_{j}(z(t))x^{T}(t)\times [(A_{i}-B_{i}F_{j}{)}^{T}P+P(A_{i}-B_{i}K_{j})]x(t)\\ +2\alpha \sum_{i=1}^{r}\sum_{j=1}^{r}p_{i}(z(t))p_{j}(z(t))x^{T}(t)\mathrm{Px}(t)\\ =\sum_{i=1}^{r}{p_{i}}^{2}(z(t))x^{T}(t)[G_{\mathrm{ii}}^{T}P+PG_{\mathrm{ii}}]x(t)+\sum_{i=1}^{r}\sum_{i<j}2p_{i}(z(t))p_{j}(z(t))x^{T}(t)\\ \times \left[{\left(\frac{G_{\mathrm{ij}}+G_{\mathrm{ji}}}{2}\right)}^{T}P+P\left(\frac{G_{\mathrm{ij}}+G_{\mathrm{ji}}}{2}\right)\right]x(t)\\ +2\alpha \sum_{i=1}^{r}{p_{i}}^{2}(z(t))x^{T}(t)\mathrm{Px}(t)+2\alpha \sum_{i=1}^{r}\sum_{i<j}2p_{i}(z(t))p_{j}(z(t))x^{T}(t)\mathrm{Px}(t)\\ =\sum_{i=1}^{r}{p_{i}}^{2}(z(t))x^{T}(t)[G_{\mathrm{ii}}^{T}P+PG_{\mathrm{ii}}+2\alpha ]x(t)\\ +\sum_{i=1}^{r}\sum_{i<j}2p_{i}(z(t))p_{j}(z(t))x^{T}(t)\\ \times \left[{\left(\frac{G_{\mathrm{ij}}+G_{\mathrm{ji}}}{2}\right)}^{T}P+P\left(\frac{G_{\mathrm{ij}}+G_{\mathrm{ji}}}{2}\right)+2\alpha \right]x(t) \end{array}$$

If inequalities (29) and (30) hold, we have

$$\overset{\cdot }{V}(x(t))+2\alpha V(x(t))<0$$

That is,

$$\overset{\cdot }{V}(x(t))<-2\alpha V(x(t))$$

Similarly, the left and right of inequalities (29) and (30) are multiplied by $P^{-1}$ , respectively. Let $X=P^{-1}$ and $Q_{i}=K_{i}X$ , the following LMI conditions including decay rate α can be obtained

(31) $$X{A_{i}}^{T}+A_{i}X-{Q_{i}}^{T}{B_{i}}^{T}-B_{i}Q_{i}+2\alpha X<0$$

(32) $$X{A_{i}}^{T}+A_{i}X+X{A_{j}}^{T}+A_{j}X-{Q_{i}}^{T}{B_{j}}^{T}-B_{i}Q_{j}-{Q_{j}}^{T}{B_{i}}^{T}-B_{j}Q_{i}+4\alpha X\leq 0$$

The unknown matrix *X* and $Q_{i}$ can be solved by the LMIs in (31) and (32). The gains $K_{i}$ and matrix *P* are calculated as below

(33) $$K_{i}=Q_{i}X^{-1},P=X^{-1}$$

## Simulation research

**Figure 3.**
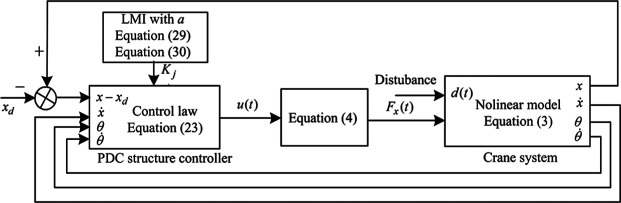
Overall block diagram of the control system.

To test the control performance of this method, the simulation research is done in MATLAB/Simulink environment. The overall block diagram of the control system is shown in Figure 3.

In the simulation, the desired position of the trolley is $x_{d}=0.6\;m$ . To test the performance of rejected disturbance, a pulse perturbation d(t) with an amplitude of 1.5 N is added when the system is stable. The system parameters are selected as follows

$$m=4\;\mathrm{kg},l=0.75\;m,M=8.5\;\mathrm{kg},\mu =0.2,g=9.8\;\mathrm{kg}/s^{2}.$$

The constraints $-\frac{\pi }{3}(\mathrm{rad})\leq \theta (t)\leq \frac{\pi }{3}(\mathrm{rad})$ and $-\frac{\pi }{4}(\mathrm{rad}/s)\leq \overset{\cdot }{\theta }(t)\leq \frac{\pi }{4}(\mathrm{rad}/s)$ are given.

Subsystem coefficient matrices of T–S model in equation (21) are determined as

$$\begin{array}{c} \begin{array}{cc} A_{1}=\left[\begin{array}{cccc} 0 & 1 & 0 & 0\\ 0 & -0.0174 & 0 & 0\\ 0 & 0 & 0 & 1\\ 0 & 0.0232 & -13.0667 & 0 \end{array}\right], & AB_{1}=\left[\begin{array}{c} 0\\ 1\\ 0\\ -1.3333 \end{array}\right]\\ A_{2}=\left[\begin{array}{cccc} 0 & 1 & 0 & 0\\ 0 & -0.0235 & 0 & 0\\ 0 & 0 & 0 & 1\\ 0 & 0.0314 & -13.0667 & 0 \end{array}\right], & B_{2}=\left[\begin{array}{c} 0\\ 1\\ 0\\ -1.3333 \end{array}\right] \end{array}\\ \begin{array}{cc} A_{3}=\left[\begin{array}{cccc} 0 & 1 & 0 & 0\\ 0 & -0.0174 & 0 & 0\\ 0 & 0 & 0 & 1\\ 0 & 0.0116 & -13.0667 & 0 \end{array}\right], & B_{3}=\left[\begin{array}{c} 0\\ 1\\ 0\\ -0.6667 \end{array}\right],\\ A_{4}=\left[\begin{array}{cccc} 0 & 1 & 0 & 0\\ 0 & -0.0235 & 0 & 0\\ 0 & 0 & 0 & 1\\ 0 & 0.0157 & -13.0667 & 0 \end{array}\right], & B_{4}=\left[\begin{array}{c} 0\\ 1\\ 0\\ -0.6667 \end{array}\right] \end{array}\\ \begin{array}{cc} A_{5}=\left[\begin{array}{cccc} 0 & 1 & 0 & 0\\ 0 & -0.0174 & 0 & 0\\ 0 & 0 & 0 & 1\\ 0 & 0.0232 & -10.8060 & 0 \end{array}\right], & B_{5}=\left[\begin{array}{c} 0\\ 1\\ 0\\ -1.3333 \end{array}\right],\\ A_{6}=\left[\begin{array}{cccc} 0 & 1 & 0 & 0\\ 0 & -0.0235 & 0 & 0\\ 0 & 0 & 0 & 1\\ 0 & 0.0314 & -10.8060 & 0 \end{array}\right], & B_{6}=\left[\begin{array}{c} 0\\ 1\\ 0\\ -1.3333 \end{array}\right] \end{array}\\ \begin{array}{cc} A_{7}=\left[\begin{array}{cccc} 0 & 1 & 0 & 0\\ 0 & -0.0174 & 0 & 0\\ 0 & 0 & 0 & 1\\ 0 & 0.0116 & -10.8060 & 0 \end{array}\right], & B_{7}=\left[\begin{array}{c} 0\\ 1\\ 0\\ -0.6667 \end{array}\right],\\ A_{8}=\left[\begin{array}{cccc} 0 & 1 & 0 & 0\\ 0 & -0.0235 & 0 & 0\\ 0 & 0 & 0 & 1\\ 0 & 0.0157 & -10.8060 & 0 \end{array}\right], & B_{8}=\left[\begin{array}{c} 0\\ 1\\ 0\\ -0.6667 \end{array}\right] \end{array} \end{array}$$

The tests are divided into four cases in total and discussed in detail.

**Figure 4.**
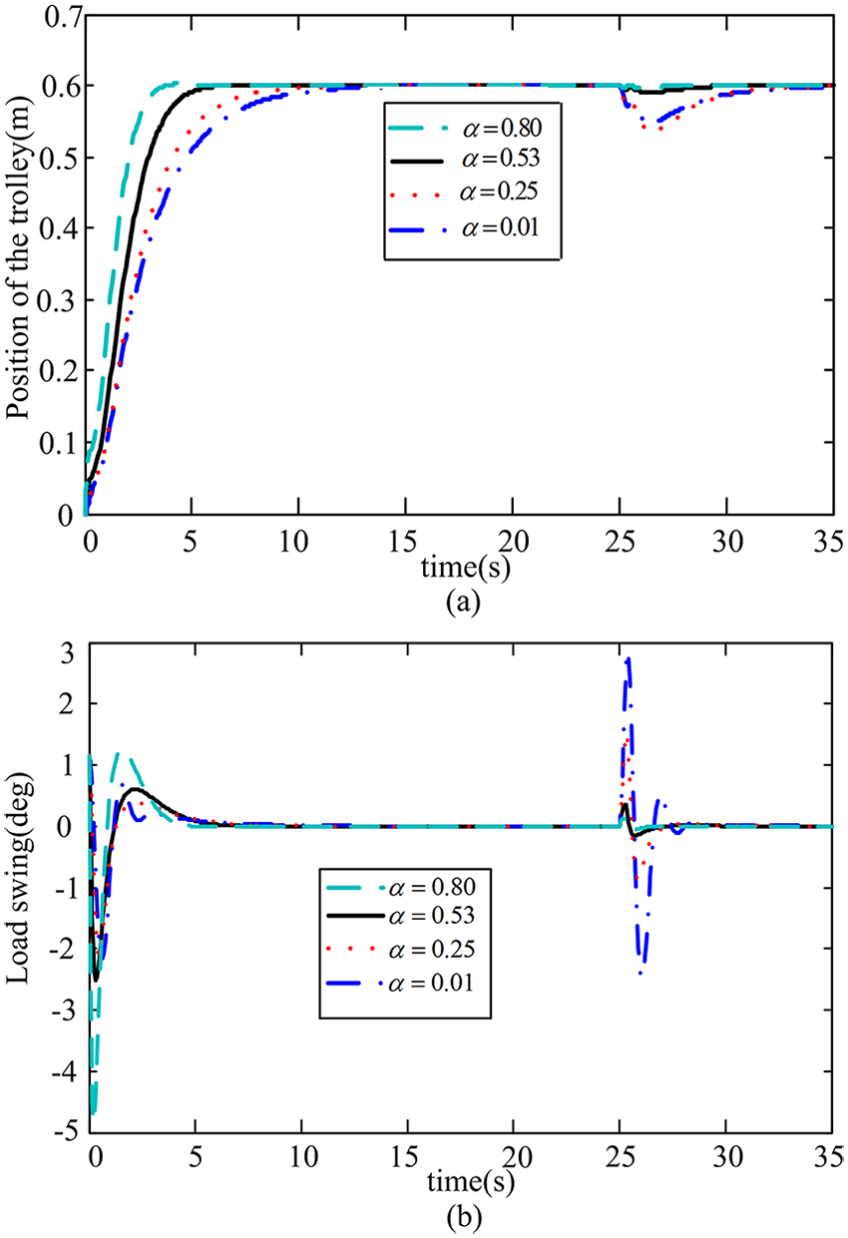
Response curve of different decay rates α . (a) Trolley position response and (b) Payload swing angle response.

*Case 1: Discussion on different decay rates α* The decay rate α affects the response speed of the system. Figure 4 shows the trolley displacement and swing angle of the payload at different α values.

As you can see from Figure 4, that the higher the decay rate α is, the faster the displacement response speed is and the stronger the rejected disturbance ability is, but the greater the payload swing angle is. After comprehensive consideration, α=0.53 is chosen in this article. Using MATLAB, the symmetric positive definite matrix *P* ensuing system stability is determined

$$P=\left[\begin{array}{cccc} 476.8731 & -282.7155 & -4.7293 & -28.8706\\ -282.7155 & 336.3575 & 33.9309 & -52.7959\\ -4.7293 & 33.9309 & 6.0796 & -12.7358\\ -28.8706 & -52.7959 & -12.7358 & 95.9853 \end{array}\right]$$

The gains $K_{j}$ are as follows

$$\begin{array}{c} K_{1}=[\begin{array}{cccc} 17.7510 & 32.9458 & -175.0808 & -1.7266 \end{array}];\\ K_{2}=[\begin{array}{cccc} 17.7554 & 32.9467 & -175.1205 & -1.7257 \end{array}]\\ K_{3}=[\begin{array}{cccc} 18.5326 & 34.7355 & -190.4480 & -4.2406 \end{array}];\\ K_{4}=[\begin{array}{cccc} 18.5275 & 34.7206 & -190.4016 & -4.2411 \end{array}]\\ K_{5}=[\begin{array}{cccc} 19.1524 & 35.4767 & -188.3394 & -1.3522 \end{array}];\\ K_{6}=[\begin{array}{cccc} 19.1548 & 35.4745 & -188.3598 & -1.3512 \end{array}]\\ K_{7}=[\begin{array}{cccc} 20.9061 & 39.0106 & -212.2236 & -3.5317 \end{array}];\\ K_{8}=[\begin{array}{cccc} 20.8061 & 38.9868 & -212.1307 & -3.5330 \end{array}] \end{array}$$

**Figure 5.**
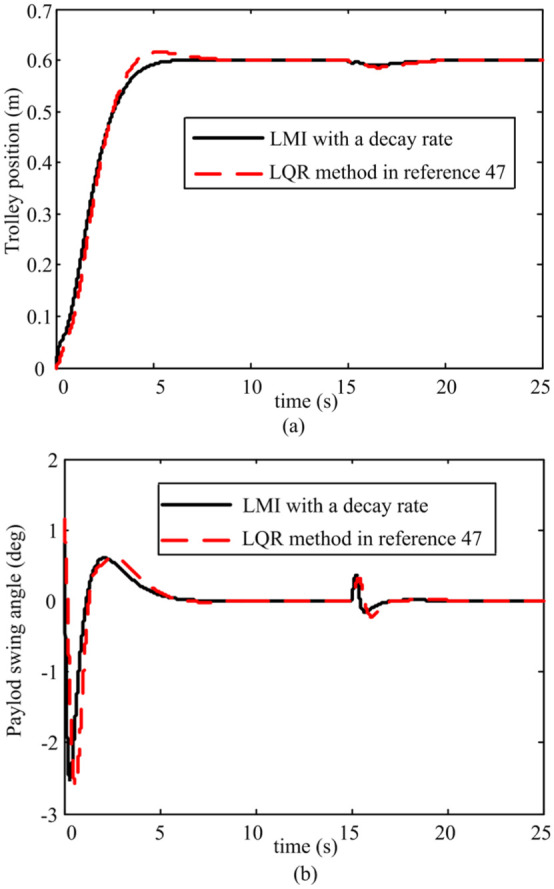
Comparison results of different control methods. (a) Trolley position response and (b) Payload swing angle response.

*Case 2: Comparison study*. First, a comparison study between LMI with a decay rate and linear quadratic regulator (LQR) method in Sun et al.^47^ is done. The simulation results are given in Figure 5.

The results clearly show that the two methods can both ensure that the system has no residual swing of the payload at the target location. However, the proposed controller in this article possesses more excellent positioning, rejected disturbance performance than the LQR method in Sun et al.^47^ The detailed quantified results of each method are summarized in Table 3.

**Table 3.** Comparison of LMI with decay and LQR method.

Process Control performance Method	Transient process under given action			Transient process under disturbance		
	Trolley running time (s)	Maximum load swing angle (°)	Overshoot (%)	Displacement change (m)	Recovery time (s)	Maximum load swing angle (°)
LMI with decay rate	7	2.535	0	0.007	3	0.351
LQR method in Sun et al^47^	9.25	2.600	2.6	0.014	5.3	0.332

LMI: linear matrix inequality; LQR: linear quadratic regulator.

As can be seen from Table 3, when LMI with decay rate is used, the trolley arrives at the desired position within 7 s, and the maximum value of swing angle is 2.535° . The trolley transportation time is 9.25 s when the LQR method is used, and the maximum value of swing angle is 2.600° . When the LMI with a decay rate and LQR method are adopted, the displacement change is 0.007 and 0.014 under the action of pulse disturbance, respectively.

Second, the control effect of the controller based on sector nonlinear model in this article and the controller based on local approximate model^48^ are compared. According to reference Zhou et al.,^48^ the nonlinear model of crane is linearized at 0° and ±45°, and the system input matrices of linearized state space model are as follows, respectively

$$\begin{array}{c} \begin{array}{cc} A_{1}=\left[\begin{array}{cccc} 0 & 1 & 0 & 0\\ 0 & -0.0235 & 4.6118 & 0\\ 0 & 0 & 0 & 1\\ 0 & 0.0314 & -19.2157 & 0 \end{array}\right], & B_{1}=\left[\begin{array}{c} 0\\ 0.1176\\ 0\\ -0.1569 \end{array}\right] \end{array}\\ \begin{array}{cc} A_{2}=\left[\begin{array}{cccc} 0 & 1 & 0 & 0\\ 0 & -0.0190 & -0.7111 & 0\\ 0 & 0 & 0 & 1\\ 0 & 0.0180 & -6.8091 & 0 \end{array}\right], & B_{2}=\left[\begin{array}{c} 0\\ 0.0952\\ 0\\ -0.0898 \end{array}\right] \end{array} \end{array}$$

Using LMIs, the gain matrices of reference Zhou et al.^48^ can be obtained

**Figure 6.**
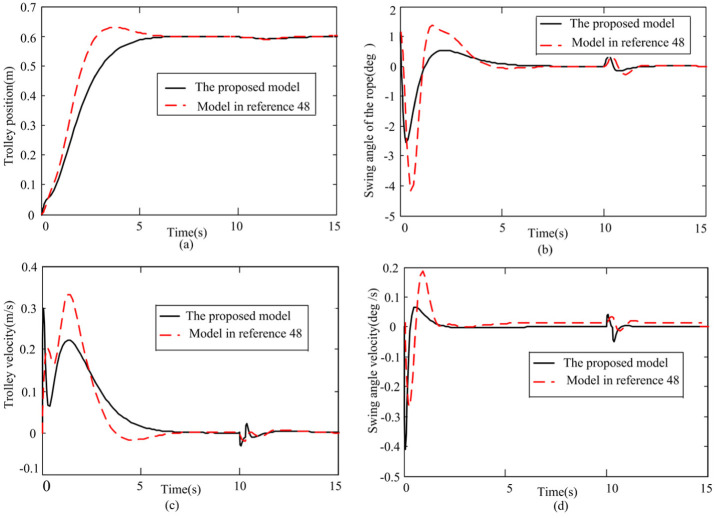
Comparison of two different models. (a) Trolley position response, (b) Swing angle response, (c) Trolley velocity response, and (d) Swing angle velocity response.

Figure 6 gives the simulation results of displacement, velocity of the trolley and the swing angle of load. The specific indicators are shown in Table 4.

**Table 4.** Comparison of control effects based on two different models.

Process Control performance Method	Transient process under given action				Transient process under disturbance		
	Transportation time (s)	Maximum load swing angle (°)	Overshoot (%)	Trolley maximum speed (m/s)	Displacement change (m)	Recovery time (s)	Maximum load swing angle (°)
Model in this article	7	2.535	0	0.22	0.007	3	0.351
Model in reference Zhou et al.^48^	8.2	4.182	4.8	0.33	0.005	3.35	0.355

As can be seen from Figure 6 and Table 4, the rejected disturbance capability of the two methods is approximately equal, and the transient processes under the given action are different. The proposed approach enables the system to arrive at the desired position within 7 s without overshoot, the maximum load swing angle to be 2.535° and the maximum speed of the trolley to be 0.22 m/s. By contrast, the approach in Zhou et al.,^48^ allows the system to arrive at the desired position within 8.2 s, with the maximum swing angle being about 4.182° and the maximum speed being 0.33 m/s.

**Figure 7.**
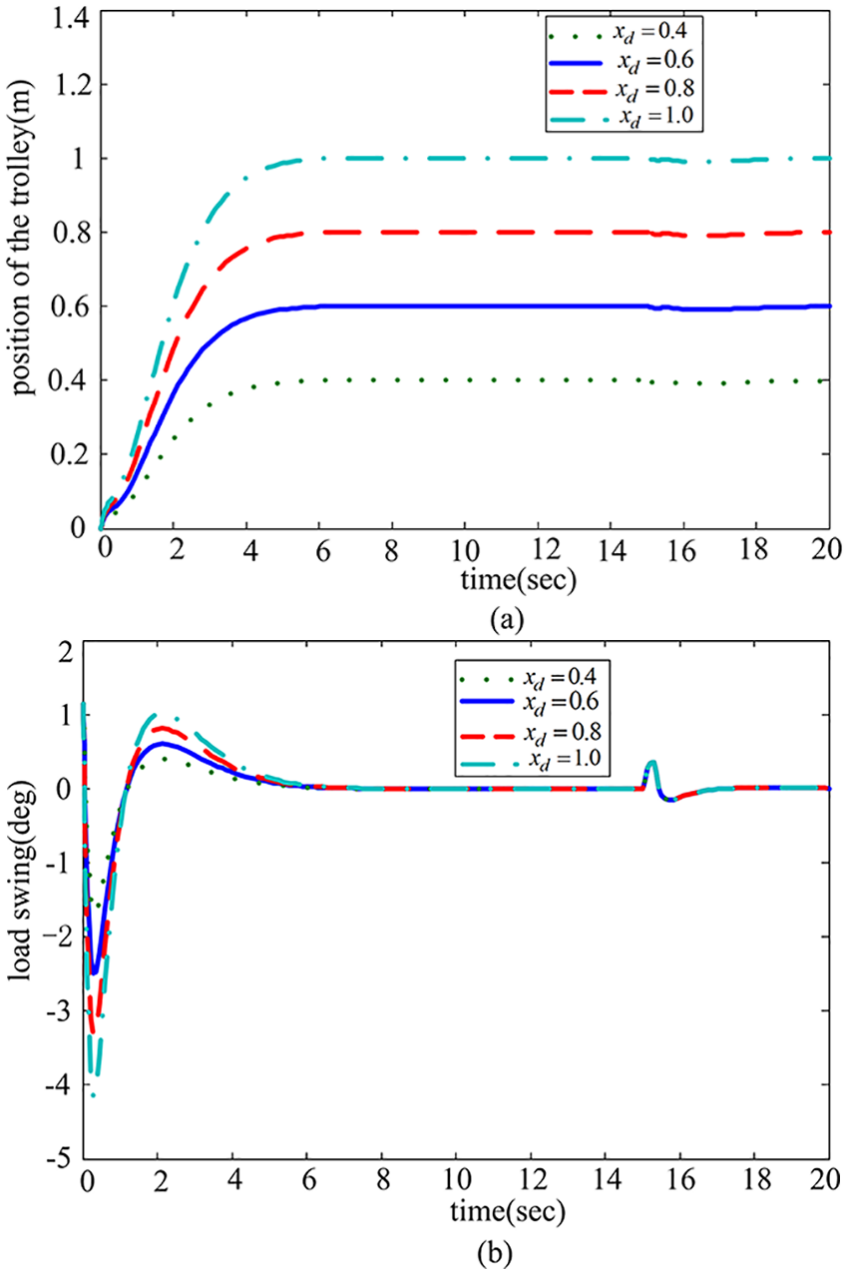
Response results with different distances $x_{d}$ . (a) Trolley position response and (b) Payload swing angle response.

*Case* 3: *Different transportation distances*. The four distances $x_{d}=0.4\;m,x_{d}=0.6,\;x_{d}=0.8\;\mathrm{and}\;x_{d}=1.0\;m$ are selected, respectively. Figure 7 shows the simulation results in four cases.

It can be seen from Figure 7 that the rejected disturbance performance does not change significantly when the desired position changes. The swing angle change is controlled between [−5°,5°] and there is no residual swing angle at the target position.

**Figure 8.**
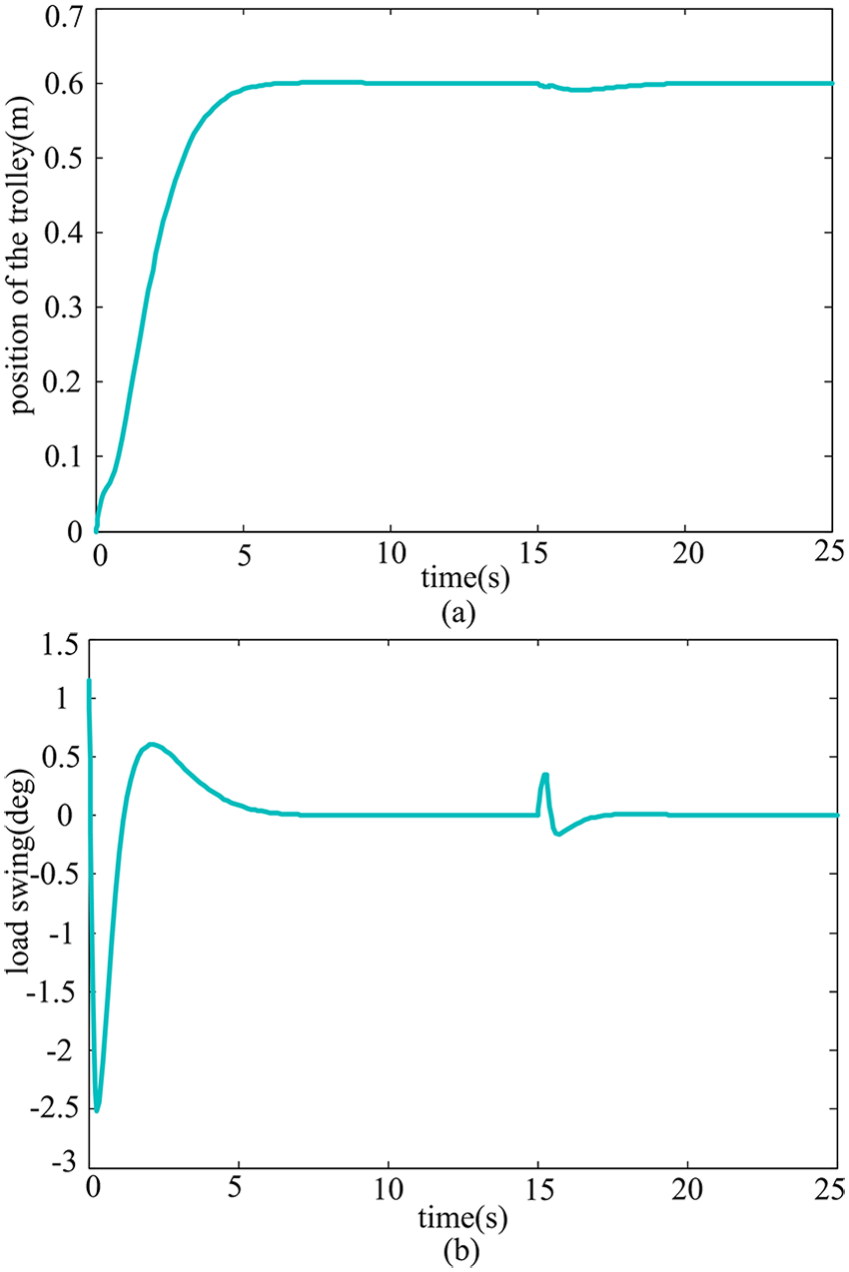
Simulation results of different trolley masses *m.* (a) Trolley position response and (b) Payload swing angle response.

**Figure 9.**
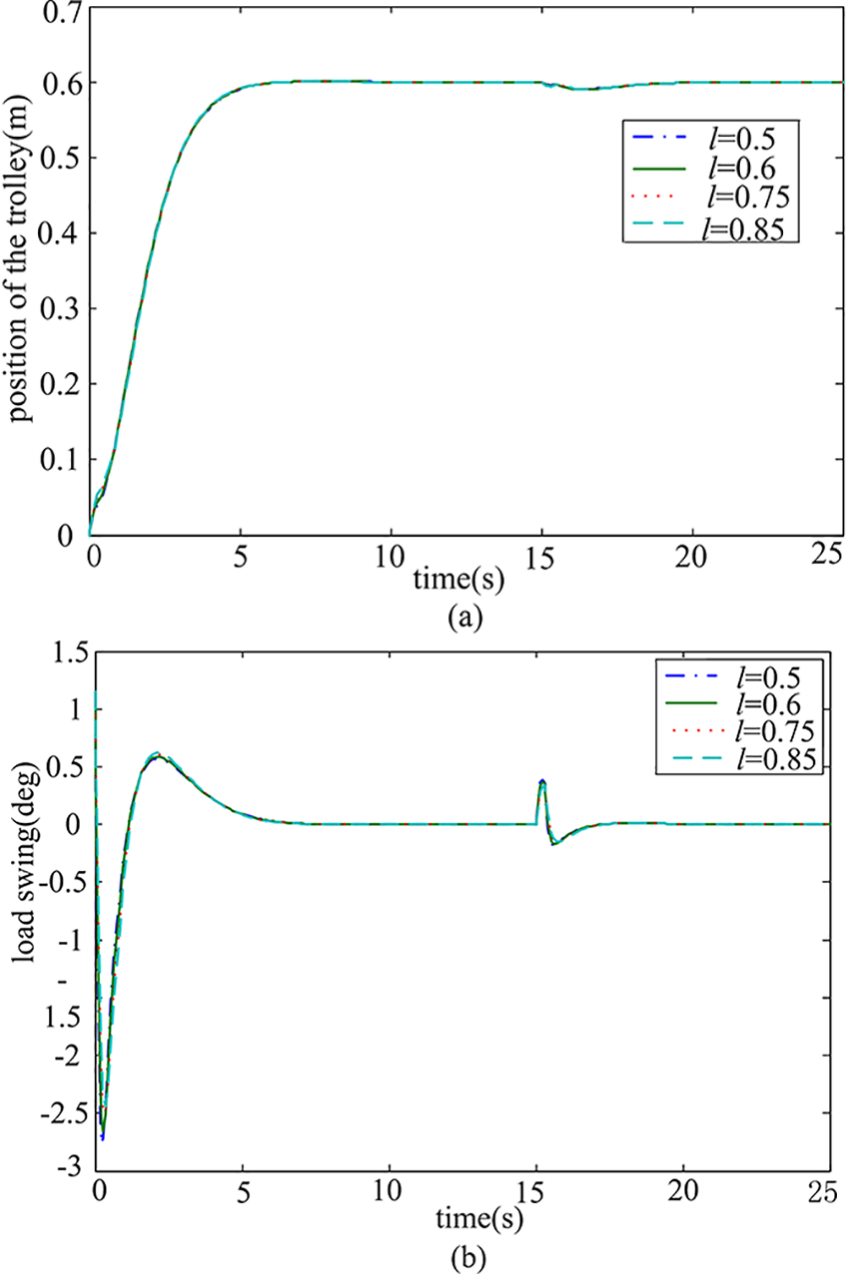
Response curve of different rope lengths *l*. (a) Trolley position response and (b) Payload swing angle response.

*Case* 4: *Robustness study of load mass and rope length variation*. In practical applications, different transportation tasks require a change in payload mass or rope length. Various payloads and rope lengths are considered in order to test the robustness of the system. In Figures 8 and 9, the simulation results are given for the variation of payload mass from 2 to 8 kg and the rope length from 0.5 to 0.85 m.

According to Figure 8, it can be seen that when the load mass *m* changes, the performance of the trolley and payload swing angle are almost unchanged. As can be seen from Figure 9, although the swing angle of the payload increases, it is controlled within the permitted range, and the rapidity of the trolley does not change much when rope length increases. It can also be seen from Figures 8 and 9 that when the payload mass and rope length change, the rejected disturbance performance does not change. The results show that the proposed method is of great significance in practical application due to its robustness against the variation of the rope length and payload mass.

## Conclusion

This study proposes a virtual control variable method to reduce the nonlinear terms in the nonlinear model of overhead cranes. The local approximate model has fewer rules, however, it cannot guarantee the global asymptotic stability of the system. On the basis of the simplified nonlinear model, the T–S fuzzy model is given using sector nonlinear technique. LMI is conservative, and the result of feedback gains calculated by LMI is unique. To improve the response speed of the system, LMI with a decay rate is considered. The proposed scheme is compared with the traditional T–S fuzzy control scheme, and the robustness of the system is discussed. The results show that the proposed scheme is feasible and effective.
